# Charge Transfer Enhancement in the D-π-A Type Porphyrin Dyes: A Density Functional Theory (DFT) and Time-Dependent Density Functional Theory (TD-DFT) Study

**DOI:** 10.3390/molecules21121618

**Published:** 2016-11-25

**Authors:** Guo-Jun Kang, Chao Song, Xue-Feng Ren

**Affiliations:** Low Carbon Energy Institute, School of Chemical Engineering & Technology, China University of Mining & Technology, Xuzhou 221008, China; gjkang@cumt.edu.cn (G.-J.K.); schaocumt@126.com (C.S.)

**Keywords:** DSSCs, charge transfer, DFT, porphyrin

## Abstract

The electronic geometries and optical properties of two D-π-A type zinc porphyrin dyes (NCH_3_-YD2 and TPhe-YD) were systematically investigated by density functional theory (DFT) and time-dependent density functional theory (TD-DFT) to reveal the origin of significantly altered charge transfer enhancement by changing the electron donor of the famous porphyrin-based sensitizer YD2-o-C8. The molecular geometries and photophysical properties of dyes before and after binding to the TiO_2_ cluster were fully investigated. From the analyses of natural bond orbital (NBO), extended charge decomposition analysis (ECDA), and electron density variations (Δρ) between the excited state and ground state, it was found that the introduction of N(CH_3_)_2_ and 1,1,2-triphenylethene groups enhanced the intramolecular charge-transfer (ICT) character compared to YD2-o-C8. The absorption wavelength and transition possess character were significantly influenced by N(CH_3_)_2_ and 1,1,2-triphenylethene groups. NCH_3_-YD2 with N(CH_3_)_2_ groups in the donor part is an effective way to improve the interactions between the dyes and TiO_2_ surface, light having efficiency (LHE), and free energy change (ΔG_inject_), which is expected to be an efficient dye for use in dye-sensitized solar cells (DSSCs).

## 1. Introduction

Dye-sensitized solar cells (DSSCs) have attracted extensive attention due to their potential advantages of low cost, easy fabrication, and flexibility in comparison with conventional silicon-based photovoltaic devices [1]. In order to improve the overall device efficiency and stability, a considerable amount of research effort has been devoted to the development of new and efficient sensitizers [2,3,4,5,6,7,8,9,10]. Among them, ruthenium (Ru) sensitizers (such as the prototype N3 and N719) were reported to be the highest efficiency dyes with a conversion efficiency of more than 11.1% [11,12,13]. However, the Ru sensitizers are not only high cost, but also environmentally unfriendly; therefore, it is necessary to search for new and efficient metal-free organic dyes.

The strong and wide absorption of solar light in the 400–750 nm range is an important feature of efficient dyes for DSSCs. Therefore, D-π-A-based zinc porphyrin dyes, such as YD2-o-C8, have been synthesized and used in DSSCs. The power conversion efficiency (PCE) of YD2-o-C8 reached 12.3% [14,15]. Another key factor determining sensitizer efficiency is fast intermolecular charge-transfer (ICT) characters from the electronic donors to acceptors, which would efficiently direct the electron flow from the sensitizer toward the semiconductor surface, thus improving energy conversion efficiency. Therefore, to further improve the efficiencies of DSSCs, various porphyrin dyes are synthesized by a push-pull structure. Santhanamoorthi et al. [16] theoretically designed a series of porphyrin sensitizers with funan and thiophene in the π conjugation linker and amine as donor part. Wu et al. [17] investigated a series of porphyrin polyoxometalate hybrids to investigate the influence of π-linkers on the photophysical properties as well as photovoltaic performances. Although the approach of extended π-conjugation frameworks can achieve a panchromatic absorption and a high Jsc [18], the Voc might be normally decreased due to the unavoidable aggravated dye aggregation [19,20].

**Figure 1:**
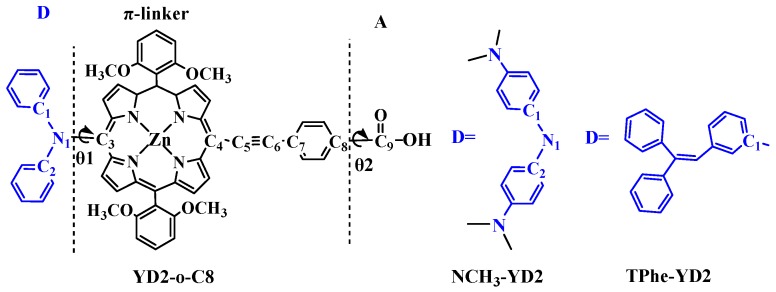
Schematic structure and the optimized geometry of YD2-o-C8, NCH_3_-YD2, and TPhe-YD2, together with the labeling scheme. The D, π-linker, and A indicates donor, π, and acceptor parts, respectively.

To obtain efficient dyes with proper donor, NCH_3_-YD2 is designed by introducing N(CH_3_)_2_ groups at the donor part of YD2-o-C8 (Figure 1), because N(CH_3_)_2_ groups were proven to be useful for the improvement of the optical properties of dyes in our previous work [21]. Since 1,1,2-triphenylethene is a good donating part in the famous D149 dye [22], it is anticipated that the efficiency of TPhe-YD (Figure 1) might be improved by using it as the electron donor. The electronic structures, optical properties, ICT, driving force of electron injection (ΔG_inject_), and light harvesting efficiency (LHE) of these dyes were fully analyzed by using density functional theory (DFT) and time-dependent density functional theory (TDDFT). The calculated results will provide an in-depth understanding of the nature of the ICT, providing useful information for the design and synthesis of porphyrin dyes for application in DSSCs.

## 2. Results and Discussion

### 2.1. Geometric Structures of the Dyes

The schematic structures of the studied dyes are drawn in Figure 1. YD2-o-C8 is composed of diphenylamine donor part (D), porphyrin derivatives (π-linker), and carboxylic acid (A), as depicted in Figure 1. NCH_3_-YD2 and TPhe-YD2 are obtained by adding N(CH_3_)_2_ and 1,1,2-triphenylethene groups in the donor part of YD2-o-C8, respectively.

The bond lengths between the D and π-linker as well as between the A and π-linker are important to serve as the bridge of the intermolecular charge transfer (ICT). The smaller bond lengths of the bridging bond will benefit the ICT in D-π-A molecules. Table 1 shows the calculated important bond lengths (Å) of these dyes. The bond lengths in the π-linker and A part in NCH_3_-YD2 and TPhe-YD2 show similar values with those in YD2-o-C8. A distinct feature is the bond length between the D and π-linker. The N1-C3 of NCH_3_-YD2 (1.431 Å) is much smaller than those of YD2-o-C8 (1.437 Å) and TPhe-YD2 (1.496 Å). This means that NCH_3_-YD2 is favorable for the electron transfer from the donor part to the π-linker. Normally, the degree of conjugation serves as a crucial factor affecting the performance of the dyes. Therefore, the dihedral angles between the D part and the π-linker (θ1), as well as between the π-linker and A (θ2) of studied dyes are collected in Table 1. As listed in Table 1, the dihedral angles θ1 of YD2-o-C8, NCH_3_-YD2, and TPhe-YD2 are −71.3°, −69.3°, and 64.9°, respectively, which is useful to hamper the dye aggregation. In addition, the dihedral angles θ2 of YD2-o-C8, NCH_3_-YD2, and TPhe-YD2 are about 0.0°, indicating that the charge is favorable to transfer from the π-linker to the A part from the geometrical point of view.

### 2.2. Intermolecular Charge Transfer

To reveal the origin of the electron transfer mechanism, the natural bond orbital analysis of studied dyes in the ground state (S_0_) and excited state (S_1_) state have been calculated and collected in Table 2. The positive charges of the donor and π-linker indicate that they are an effective electron-pushing unit, whereas the negative charges on the A acceptors demonstrate that it is useful to trap the electron. As listed in Table 2, in the S_0_ state, the atomic charge on the donor of YD2-o-C8 is −0.181 e, indicating that diphenylamine is not an effective electron-pushing unit in the dyes. The increased positive charge of the D part of NCH_3_-YD2 (−0.139 e) relative to YD2-o-C8 (−0.181 e) indicates that the electrons on the donor part are easily transferred to the π-linker by introducing the N(CH_3_)_2_ substitutions. Therefore, the electrons of NCH_3_-YD2 are more effectively transferred to the acceptor (−0.007 e) relative to YD2-o-C8 (−0.004 e). With respect to TPhe-YD2, although the donor part is positively charged (0.007 e), the electrons on the π-linker are small, indicating that TPhe-YD2 is not an effective electron-pushing unit. Furthermore, comparing the ground state (S_0_) with the excited state (S_1_) state, the A group in YD2-o-C8, TPhe-YD2, and NCH_3_-YD2 is negatively charged, which suggests that the electrons can be promoted to excited states by the absorption of light energy.

To be more quantitative, charge decomposition analysis (CDA) and extended charge decomposition analysis (ECDA) of these dyes were carried out to develop an understanding of the process of the electron transfer mechanism. The d denotes the amount of donated electron from the D part to the (A + (π-linker)) part. On the contrary, b denotes electrons that were back donated from the A part to the (D + (π-linker)) unit. The difference between d and b (d–b) means the total number of donation and back donation electrons of the studied dyes [23,24]. The calculated d–b results are also collected in Table 2. As listed in Table 2, all the d–b data of these dyes are positive values, indicating that electrons should transfer from the (D + (π-linker)) part to the A unit. Furthermore, the ECDA is 0.2005 for YD2-o-C8, indicating that the amount of electron transfer from the (D + (π-linker)) unit to the A part is 0.2005. As expected, the amount of electron transfer for NCH_3_-YD2 (0.2047) is much larger than that of YD2-o-C8. Therefore, it can be inferred that the introduction of N(CH_3_)_2_ groups is favorable to enhance the intermolecular charge transfer.

**Figure 2:**
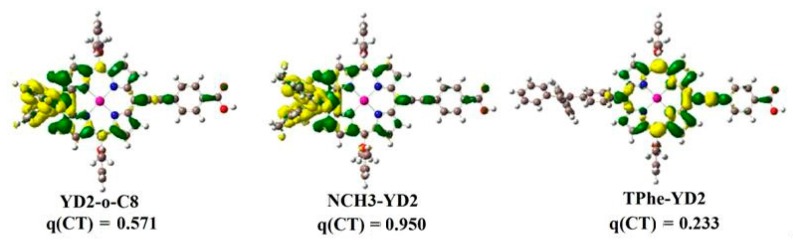
The electron density variation (Δρ) between the excited state and ground state. The green color indicates increased electron density, while yellow color indicates decreased electron density. q(CT) is the amount of charge transfer (e-).

Furthermore, the electron density variations (Δρ) between the excited state and ground state of studied dyes are also explored and depicted in Figure 2. For YD2-o-C8 and NCH_3_-YD2, the decreased electron density (yellow color) is mainly localized on the donor and the porphyrin linker, while the increased electron density (green color) is localized on the π-linker and A groups. Therefore, the electronic transition of these dyes may be involved in ICT, which allows rapid interfacial electron injection from the dye to the conduction band (CB) of TiO_2_. As shown in Figure 2, the transferred charges q(CT) of studied dyes increase in the following order: TPhe-YD2 (0.233 eV) < YD2-o-C8 (0.571 eV) < NCH_3_-YD2 (0.950 eV), suggesting that the ICT process may be greatly enhanced by the introduction of N(CH_3_)_2_ in the donor part of YD2-o-C8.

### 2.3. Frontier Molecular Orbitals

To reveal the photophysical properties of these dyes, the energy levels of frontier molecular orbitals, as well as the HOMO–LUMO energy gap (Eg) of these dyes are collected in Table 3. The calculated HOMO (−4.68 eV), HOMO − 1 (−5.04 eV), LUMO (−2.34 eV), and LUMO + 1 energy levels (−2.01 eV) of YD2-o-C8 give a value close to the calculated values (−4.632, −5.004, −2.298, −1.967 eV, respectively) [14]. Furthermore, the energy level of HOMO of YD2-o-C8 (−4.68 eV) is close to the redox potential of Iˉ/I_3_ˉ (−4.8 eV) [25]. The introduction of 1,1,2-triphenylethene substitutions decreases the HOMO energy level relative to YD2-o-C8. Therefore, from the energetic point of view, TPhe-YD2 should be capable of getting electrons from the Iˉ/I_3_ˉ. In addition, the LUMO energy levels of these dyes are above the conduction band edge of TiO_2_ (−4.00 eV) [26], ensuring the thermodynamic driving force for electron injection from the excited state dye to the CB edge of TiO_2_. Clearly, all these dyes should be capable of injecting electrons into TiO_2_. With the dramatic enhancement of the HOMO energy level, the Eg value of the studied dyes increases in the following order: NCH_3_-YD2 (1.90 eV) < YD2-o-C8 (2.34 eV) < TPhe-YD2 (2.47 eV). This suggests that the absorption wavelength might have a blue-shifted tendency: NCH_3_-YD2 < YD2-o-C8 < TPhe-YD2 when the electron transition from HOMO to LUMO makes a large contribution to the absorption wavelength.

**Figure 3:**
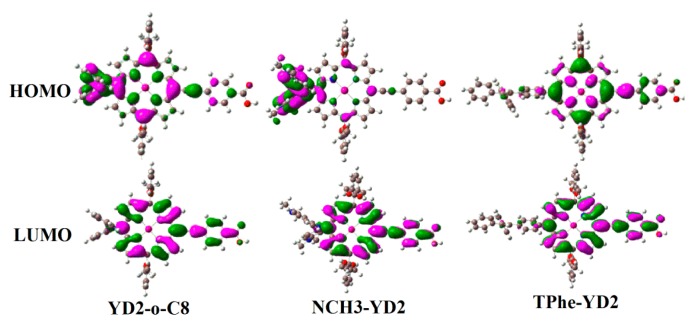
The electron density distributions for the HOMO and LUMO.

On the other hand, the composition of the frontier molecular orbital is closely related to the intermolecular charge transfer behavior. Therefore, electron density distributions for the HOMO and LUMO of the studied dyes are shown in Figure 3. As can be seen in Figure 3, the HOMO of YD2-o-C8 is mainly constructed by the (D + (π-linker)) unit, while the LUMO is constructed by the ((π-linker) + A) unit. Thus, it can be inferred that the electron transfers from the D part to the A part through the π-linker. With respect to NCH_3_-YD2, the HOMO is mainly constructed by the D unit, while LUMO consists of the ((π-linker) + A) part. Obviously, the electron transition from the HOMO to LUMO of NCH_3_-YD2 could lead to typical electron transfer from the D part to the π-linker and the A part. For TPhe-YD2, the HOMO and LUMO are composed of the ((π-linker) + A) unit, suggesting that the ICT process of TPhe-YD2 may be reduced. This is consistent with the ECDA and q(CT) analyses.

### 2.4. The Absorption Spectra

**Figure 4:**
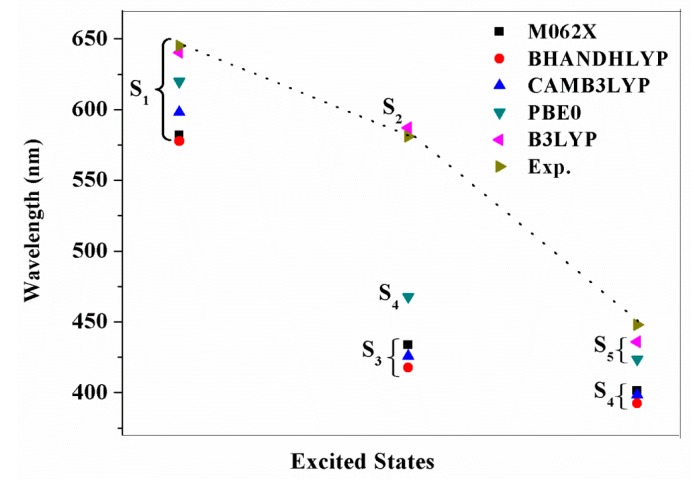
The absorption spectrum (nm) for of YD2-o-C8 obtained by M062X, BHANDHLYP, CAMB3LYP, PBE0, and B3LYP in combination with the polarized continuum model (PCM) in tetraHydroFuran (THF) medium, together with the experimental values [14].

**Figure 5:**
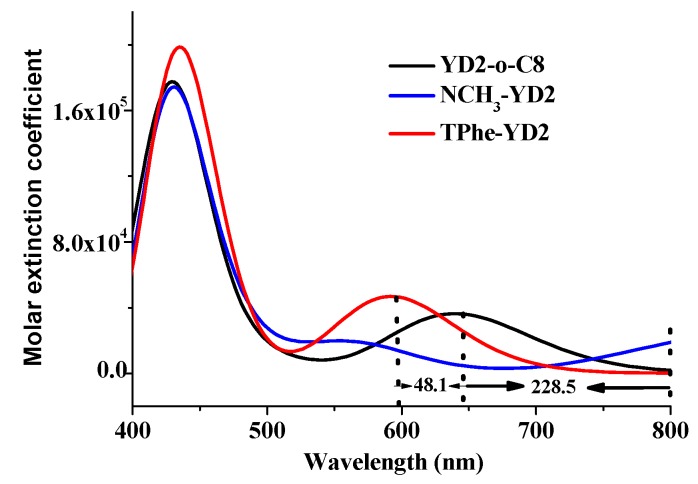
The simulated absorption spectra of studied dyes obtained by B3LYP method in THF media.

The calculated absorption spectra (nm) of YD2-o-C8 obtained by M062X, BHANDHLYP, CAMB3LYP, PBE0, and B3LYP in combination with the polarized continuum model (PCM) in THF medium are drawn in Figure 4. Figure 4 shows that the choice of different functionals has a large influence on the absorption spectra. The lowest lying absorption spectrum (S1) of YD2-o-C8 is 581.9, 577.6, 598.1, 619.7, and 640.3 nm, respectively, obtained by M062X, BH and HLYP, CAMB3LYP, PBE0, and B3LYP methods. Compared with the experimental values (645 nm) [14], it is apparent that TD-B3LYP gives a satisfactory result, while other functionals dramatically underestimate the absorption wavelength. Similarly, the maximum absorption spectrum of YD2-o-C8 obtained by the TD-B3LYP method (S5 state, at 436.0 nm) is very close to experimental data (448 nm) [14] relative to other functionals. The calculation is consistent with the previous work showing that the TD-B3LYP method is suitable for the prediction of the absorption spectra of porphyrin relatives, even with a charge transfer character. Hence, the absorption spectra of NCH_3_-YD2 and TPhe-YD2 are calculated by B3LYP functional in conjunction with the PCM model. The simulated absorption spectra for these dyes are drawn in Figure 5, and the calculated absorption spectra, oscillator strength (f), and nature of the transitions are presented in Table 4.

From Figure 5, YD2-o-C8 displays an intensity B band at 436.0 nm and a weak Q band at 640.3 nm. The changing of the electron donor part has a great effect on the absorption spectra. Compared with YD2-o-C8, the Q bands of NCH_3_-YD2 and TPhe-YD2 are dramatically red-shifted and blue-shifted by 228.5 and 48.1 nm, respectively, which agrees well with the tendency of their Eg in Section 2.3. In addition, NCH_3_-YD2 and TPhe-YD2 have excellent B bands, which can absorb light from 400 to 500 nm with high molar extinction coefficients. TPhe-YD2 exhibits better absorption strength in both B band and Q band relative to YD2-o-C8. It is anticipated that the efficiency may be improved by adding these substitutions.

**Figure 6:**
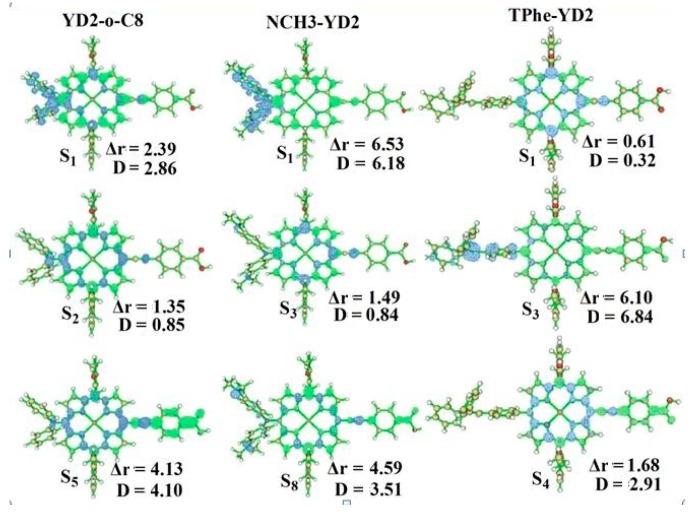
The hole and electron distributions for the studied dyes. The blue and green isosurfaces represent hole and electron distributions, respectively.

To further analyze the nature of the electronic excitation of absorption, the hole and electron distributions of these absorption peaks were calculated by Multiwfn software [23], and the calculated results for the main absorption peaks of the studied dyes are drawn in Figure 6. Δr is used as quantitative analysis of electron excitation mode. Larger Δr values indicate an increased likelihood that the transition possesses a strong CT character. D is the distance between the centroid of the hole and the electron; therefore, the larger D value suggests the transition belongs to a CT character. As drawn in Figure 6, the S_1_ and S_5_ transitions for YD2-o-C8 possess a CT character. The holes of S_1_ and S_5_ transitions are mainly contributed by the D part and π-link, respectively, while the electrons of these transitions are only located on the A unit. Furthermore, according to the analyses of Δr and D, it can be concluded that the S_1_ and S_5_ transitions for YD2-o-C8 belong to CT type. Furthermore, the Δr and D values of S_1_ and S_8_ transitions for NCH_3_-YD2 become much larger than the peaks of YD2-o-C8. Hence, the modifications of the donor part have been adopted as an efficient way to improve the optical properties.

### 2.5. Structures and Properties of Dyes/TiO_2_ Complexes

**Figure 7:**
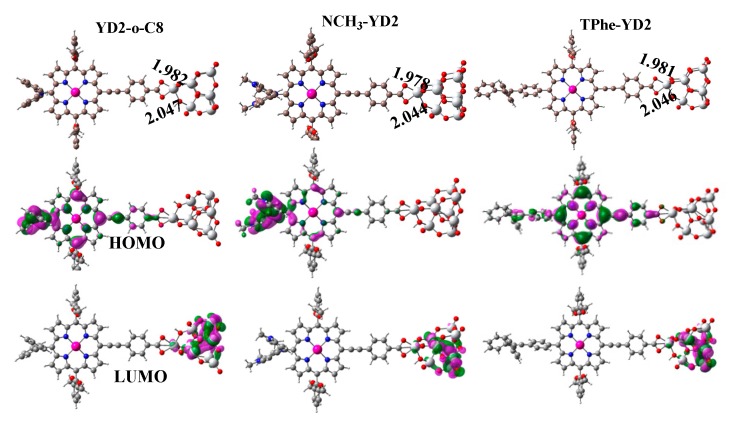
The optimized molecular structures (Å) and electron density distributions for the HOMO and LUMO orbitals of dyes/TiO_2_ complexes.

To gain insight into the electronic structure of the dye/TiO_2_ interface, we consider the dye/(TiO_2_)_9_ system with bidentate chelating configuration absorption modes as our simulation model, which is the most stable absorption model [27,28]. The optimized geometries of the dye/(TiO_2_)_9_ complexes are drawn in Figure 7, together with the important bond lengths and the electron density distributions for the HOMO and LUMO. Table 5 presents the absorption energies, HOMO and LUMO energies, and ECDA.

As shown in Figure 7, the two carboxylate oxygens and titanium atoms on the dye-TiO_2_ interfaces are 1.982 and 2.047 Å for YD2-o-C8/(TiO_2_)_9_, 1.978 and 2.044 Å for NCH_3_-YD_2_/(TiO_2_)_9_, and 1.981 and 2.046 Å for TPhe-YD_2_/(TiO_2_)_9_. As reported in previous studies [29,30], there is a strong interaction between the dye and the TiO_2_ surface when Ti-O lengths are less than 2.1 Å. Hence, it is expected that these dyes bind tightly to the TiO_2_ surface. Furthermore, Table 5 shows that the absorption energy (E_ads_) of these dye/(TiO_2_)_9_ is calculated to be ca. 65 kcal/mol, which is sufficiently large to allow the assumption that these dyes can be chemisorbed on the (TiO_2_)_9_. The E_ads_ values of NCH_3_-YD2/(TiO_2_)_9_ and TPhe-YD2/(TiO_2_)_9_ are slightly increased by 0.74 and 0.25 kcal/mol, respectively, compared with YD2-o-C8/(TiO_2_)_9_, suggesting that the interactions between the dyes and the TiO_2_ surface are enhanced by the modification of the donor part by the N(CH_3_)_2_ and the 1,1,2-triphenylethene group in the donor part of YD2-o-C8.

Compared with the corresponding isolated dyes, the HOMO energy levels of YD2-o-C8/(TiO_2_)_9_, NCH_3_-YD2/(TiO_2_)_9_, and TPhe-YD2/(TiO_2_)_9_ are increased by 0.39, 0.32, and 0.21 eV, respectively. Furthermore, the LUMO of YD2-o-C8/(TiO_2_)_9_ is 1.57 eV lower after binding to the (TiO_2_)_9_ surface. For NCH_3_-YD2/(TiO_2_)_9_ and TPhe-YD2/(TiO_2_)_9_, their LUMOs are calculated to be 1.65 and 1.63 eV lower after binding to the (TiO_2_)_9_ surface. This indicates that there is a stronger electron coupling between the designed dyes and the (TiO_2_)_9_ surface compared with the experimental dyes. Besides, the HOMOs of the dye/(TiO_2_)_9_ are the π-orbital that is delocalized over the dyes, while the LUMOs are π*-orbital localized on the (TiO_2_)_9_. This means that the HOMO to LUMO transition of these dye/TiO_2_ complexes possess a CT character. To be more quantitative, the ECDA of NCH_3_-YD2/(TiO_2_)_9_ are explored and listed in Table 5. As expected, the ECDA of NCH_3_-YD2/(TiO_2_)_9_ (0.2670) exhibits a slightly larger value with respect to its reference dye YD2-o-C8/(TiO_2_)_9_ (0.2541) and TPhe-YD2/(TiO_2_)_9_ (0.2559), indicating that the charge transfer is most favorable for TPhe-YD2/(TiO_2_)_9_.

### 2.6. Photoelectric Conversion Efficiency of Dyes

It is desired to have rapid and efficient electron injection from the dyes to the TiO_2_ surface. To quantify the electron injection, the free energy change (ΔG_inject_) and light-harvesting efficiency (LHE) at a given wavelength are calculated by the following equation [31]:

(1) $$\Delta G_{\text{inject}}=E_{OX}^{dye*}-E_{CB}^{SC}$$

(2) LHE = 1 − 10^−f^

where f is oscillator strength of the maximum absorption spectra, $E_{CB}^{SC}$ is the reduction potential of the CB of the semiconductor (4.00 eV) [26], and $E_{OX}^{dye*}$ is the oxidation potential of the dye in the excited state, which can be obtained by

(3) $$E_{OX}^{dye*}=E_{OX}^{dye}-\lambda_{\text{max}}$$

where $E_{OX}^{dye}$ is the redox potential of ground state and λ_max_ is the vertical transition energy. The calculated $E_{OX}^{dye*}$, $E_{OX}^{dye}$, ΔG_inject_, and LHE of these dyes are listed in Table 6. It can be found that the LHE is not sensitive to the change of the donor part. With respect to ΔG_inject_, all the calculated results are very negative, especially for NCH_3_-YD2 (−2.35 eV), indicating that the dye’s excited state lies above the TiO_2_ conduction band edge, therefore favoring the electron injection from the dyes to the TiO_2_ surface. Thus, from the calculations of ΔG_inject_ and LHE, NCH_3_-YD2 is very promising to provide better performance as a sensitizer in DSSCs.

## 3. Computational Details

The ground-state geometries of all sensitizers before and after binding to TiO_2_ were fully optimized using DFT at the B3LYP [32,33] level with the LANL2DZ basis set for metal atoms and the 6-31G(d) basis set for non-metal atoms. The B3LYP method has been reported as a reliable method of calculating the geometry of YD2-o-C8 [34,35,36]. Vibrational frequencies were further calculated at the same level to confirm that each ground geometry was a minimum on the potential energy surface. The lowest singlet excited states (S_1_) geometry of the studied complexes was calculated using the TD-B3LYP method with the LANL2DZ basis set for metal atoms and the 6-31G(d) basis set for non-metal atoms. At the same level of theory, natural bond orbital (NBO) analysis was carried out. To validate a reliable method for the prediction of vertical excitations, the absorption spectra of YD2-o-C8 was calculated by five different exchange-correlation functionals (B3LYP, CAMB3LYP [37], BHANDHLYP [38], PBE0 [39], M062X [40]) associated with the polarized continuum model (PCM) in tetraHydroFuran (THF) media. The (TiO_2_)_9_ cluster has been found to be large enough to reproduce the electronic absorption spectra and anchoring modes of TiO_2_ [41,42,43]. Additionally, the compositions and energies of HOMO and LUMO of the (TiO_2_)_9_ cluster were approximately similar to the valence band edge and conduction band edge of bulk TiO_2_ [44]. Therefore, the (TiO_2_)_9_ cluster was accepted to analyze the electronic structure and optical properties of dye-TiO_2_ systems. The structure of the (TiO_2_)_9_ cluster was optimized by the B3LYP method using the 6-31G(d) basis set for the O atom and the LANL2DZ basis set for the Ti atom. After optimization, the absorption energies (*E_ads_*) of the dyes on the (TiO_2_)_9_ cluster were calculated by the following equations:

(4) $$E_{ads}=E_{dye}+E_{{\left(TiO_{2}\right)}_{9}}-E_{dye-{\left(TiO_{2}\right)}_{9}}$$

where *E_dye_* and *E*_*dye−(TiO*2*)*9_ are the total energies of dyes before and after binding the (TiO_2_)_9_ cluster, respectively, while *E*_*(TiO*2*)*9_ is the total energy of the (TiO_2_)_9_ cluster. All of the calculations were performed by the Gaussian09 software package [45]. To gain insight into the electron transfer mechanism, the charge decomposition analysis (CDA), extended charge decomposition analysis (ECDA), and the electron density variations (Δρ) between the excited state and ground state of dyes and dye-TiO_2_ systems were calculated by Multiwfn software [23].

## 4. Conclusions

The electronic structures, optical properties, and charge transfer character for a series of porphyrin dyes have been investigated using density functional theory (DFT) and time-dependent density functional theory (TDDFT). The effects of N(CH_3_)_2_ and 1,1,2-triphenylethene on the photophysical properties are fully demonstrated. From the analyses of geometry, natural bond orbital (NBO), extended charge decomposition analysis (ECDA), and frontier molecular orbital distribution, as well as electron density variation (Δρ) between the excited state and ground state, it was found that NCH_3_-YD2 and TPhe-YD are efficient D-π-A dyes with an enhanced intermolecular charge transfer (ICT) transition. NCH_3_-YD2 has a large light having efficiency (LHE), free energy change (ΔG_inject_), and better electron transfer between the dyes and the TiO_2_ surface. This study is expected to provide useful information for the design and synthesis of porphyrin dyes for application in DSSCs.

## Figures and Tables

**Table 1:** The important bond length (Å) and torsion angle between donor and π-linker (θ1°) and π-linker and acceptor (θ2°) of studied dyes.

Molecule	π-Linker				A	Torsion Angle (°)	
	N1/C1–C3	C4–C5	C5–C6	C6–C7	C8–C9	θ1	θ2
YD2-o-C8	1.437	1.421	1.220	1.419	1.482	−71.3	0.0
NCH_3_-YD2	1.431	1.420	1.220	1.419	1.481	−69.3	0.1
TPhe-YD2	1.496	1.421	1.220	1.419	1.482	64.9	0.0

**Table 2:** The natural bond orbital (NBO) charge (e) of studied complexes in the ground state (S_0_) and excited state (S_1_) state, as well as the charge decomposition analysis (CDA) and extended charge decomposition analysis (ECDA) analyses.

Molecule	S_0_/NBO			S_1_/NBO			CDA	ECDA
	D	π-Linker	A	D	π-Linker	A	d–b	
YD2-o-C8	−0.181	0.185	−0.004	−0.185	0.193	−0.008	0.056084	0.2005
NCH_3_-YD2	−0.139	0.146	−0.007	−0.183	0.194	−0.011	0.057193	0.2047
TPhe-YD2	0.007	−0.002	−0.005	0.010	−0.002	−0.008	0.057470	0.2019

**Table 3:** The calculated frontier molecular orbital energies (eV) and HOMO-LUMO energy gap (Eg, eV) of dyes, together with available calculated values [14].

Molecule	HOMO − 1	HOMO	LUMO	LUMO + 1	Eg
YD2-o-C8	−5.04	−4.68	−2.34	−2.01	2.34
Ref. [14]	−5.004	−4.632	−2.298	−1.967	2.334
NCH_3_-YD2	−4.78	−4.08	−2.18	−1.86	1.90
TPhe-YD2	−4.98	−4.73	−2.26	−1.95	2.47

**Table 4:** The calculated absorption spectra, oscillator strength (f), and nature of the transitions, together with available experimental values [14].

Molecule	Excited States	Wavelength λ/nm (f)	Assignment Composition	Expermental [14] λ/nm (ε/10^3^ M^−1^·cm^−1^)
YD2-o-C8	S_1_	640.3 (0.4996)	0.67 H → L	645 (31)
	S_2_	587.3 (0.0028)	0.56 H → L + 1 −0.42 H − 1 → L	581 (12)
	S_5_	436.0 (1.6381)	0.49 H – 1 → L + 1 −0.40 H → L + 2	448 (212)
NCH_3_-YD2	S_1_	868.8 (0.3346)	0.70 H → L	
	S_3_	566.4 (0.2293)	0.63 H − 1 → L	
	S_8_	429.3 (1.4459)	0.53 H − 3 → L + 1	
TPhe-YD2	S_1_	592.2 (0.6461)	0.64 H → L	
	S_3_	452.7 (0.5685)	0.61 H − 2 → L	
	S_4_	432.7 (1.4308)	0.45 H − 1 → L −0.32 H − 2 → L	

H and L stands for HOMO and LUMO, respectively.

**Table 5:** The absorption energies (E_ads_), HOMO and LUMO energies, ECDA, and q(CT) of the studied dyes.

Molecule	E_ads_ (kcal/mol)	HOMO (eV)	LUMO (eV)	ECDA	q(CT) (e^−^)
YD2-o-C8	64.40	−5.07	−3.91	0.2541	2.34
NCH_3_-YD2	65.13	−4.40	−3.83	0.2670	1.90
TPhe-YD2	64.65	−5.19	−3.89	0.2559	2.72

**Table 6:** Calculated $E_{OX}^{dye}$, $E_{CB}^{SC}$, ∆G_inject_ (in eV), and light-harvesting efficiency (LHE) for the studied dyes.

Molecule	λ_max_	$E_{OX}^{dye}$	$E_{CB}^{SC}$	∆G_inject_	LHE	f
YD2-o-C8	2.8438	2.26	5.10	−1.74	0.98	1.6396
NCH_3_-YD2	2.8878	1.65	4.54	−2.35	0.96	1.4459
TPhe-YD2	2.8657	2.30	5.17	−1.70	0.96	1.4308
